# Calcium-Related Genes Predicting Outcomes and Serving as Therapeutic Targets in Endometrial Cancer

**DOI:** 10.3390/cells11193156

**Published:** 2022-10-08

**Authors:** Ting Huang, Xuan Feng, Jiaqi Wang, Jingyi Zhou, Jianliu Wang

**Affiliations:** Department of Obstetrics and Gynecology, Peking University People’s Hospital, Beijing 100044, China

**Keywords:** endometrial cancer, calcium-related gene, prognostic signature, CACNA2D1, calcium channel blocker

## Abstract

Endometrial cancer (EC) is the most common gynecologic cancer with increasing incidence. The dysregulation of intracellular calcium plays a crucial role in cancer progression. However, the relationship between calcium-related genes and prognosis remains unclear. In this study, we aimed to establish a risk model based on calcium-related genes for prognosis prediction in patients with EC. The TCGA-total set was divided into a training set and a testing set (1:1). The four-gene prognostic signature (*CACNA2D1*, *SLC8A1*, *TRPM4* and *CCL2*) was established and classified all EC patients into a low-risk or high-risk group. This model was validated in both the testing dataset and the total set. The EC patients with high RiskScores showed significantly shorter overall survival than those with low RiskScores, and this trend was consistent among most subgroups. Moreover, an enrichment analysis confirmed that calcium-related and estrogen-response signalings were significantly enriched in the high-risk group. The knockdown of *CACNA2D1* by siRNA or its blocker, amlodipine (AM) inhibited cell proliferation and induced cycle arrest in vitro. The calcium channel blocker AM inhibited cell proliferation and induced cycle arrest in vitro. AM also showed marked tumor inhibition effects in vivo. In summary, the prognostic model constructed by four calcium-related genes can reliably predict the outcomes of EC patients, and a calcium channel blocker, AM, has significant potential for EC treatment.

## 1. Introduction

Endometrial cancer (EC) is one of the most common gynecologic cancers worldwide, accounting for an estimated 7% of all female malignancies [1]. Despite significant advances in the treatment of EC, the incidence and mortality are increasing annually. In addition, the five-year survival rate for EC has not improved significantly [2]. Therefore, it is urgent to explore effective biomarkers of EC and find potential anticancer drugs.

Calcium ions are ubiquitous second messengers that regulate a wide range of cellular processes, including cell proliferation, apoptosis, migration and differentiation [3,4]. Calcium ions are reported to be involved in the genesis and development of EC. On one hand, approximately 80% of ECs are estrogen-dependent carcinomas. Estrogen can rapidly induce calcium mobilization and affect the expression of some calcium-related proteins in normal endometrial epithelial cells after long-term stimulation [5]. On the other hand, our previous analysis of clinical data showed that high calcium levels were associated with advanced stage, grade, lymph node metastasis (LNM), positive ascites cytology and lymph-vascular space invasion (LVSI), suggesting that calcium ions promote EC progression [6].

Calcium ions are imported into cells through calcium channels or exchangers on the plasma membrane [7]. Calcium voltage-gated channel subunit alpha1 D (CACNA1D) and transient receptor potential vanilloid 4 (TRPV4) affect the proliferation and metastasis of EC cells [6,8]. Solute carrier family 8, member 1 (SLC8A1), encoding the Na^+^/Ca^2+^ exchanger, has been found to regulate the traction force and promote the migration of endometrial cancer cells [9]. Given the involvement of Ca^2+^ signaling in cancer progression, calcium channels and exchangers are proposed as targets for anticancer therapeutics. Calcium channel blockers (CCBs) are commonly used to treat hypertension, acting by blocking voltage-gated calcium channels and a subsequent extracellular calcium influx. However, there is no relevant report exploring the role of calcium-related genes in predicting the prognosis of EC and the possibility of repurposing CCBs for EC treatment.

Herein, 392 calcium-related genes were collected and intersected with differentially expressed genes (DEGs) obtained from the TCGA-training set. Subsequently, we constructed a risk score model and tested its specificity and accuracy in the TCGA-testing set and TCGA-total set. Next, a gene ontology (GO) analysis and gene set enrichment analysis (GSEA) were carried out between the low-risk and high-risk groups to investigate the possible pathways related to calcium signaling. Moreover, we explored the anticancer effects of amlodipine (AM), one of the most commonly used CCBs in vitro and in vivo. Our risk score model provides insight into novel predictors and treatments for EC.

## 2. Materials and Methods

RNA sequencing expression data and clinical follow-up information from patients with UCEC were downloaded from the public TCGA database (https://portal.gdc.cancer.gov/, accessed on 1 May 2022). We removed samples without complete follow-up information; thus, 537 samples were collected in our study. In addition, 23 normal samples were included in this study. Based on a random number generator, we divided the samples into a training set and a testing set (sample size, training set:testing set = 1:1). The training set was used to develop prognostic models and screen survival-associated calcium-related genes, and the testing set was used to validate the results. The cohort information is shown in Table 1. There was no significant difference among the training set, testing set and total set in age distribution, follow-up time, histological type, stage and grade.

### 2.1. Identification of Calcium-Related Genes and Functional Analysis

The list of calcium-related genes was obtained from GeneCards, and genes with a relevance score ≥ 8 were selected. The limma R package was used to identify DEGs between normal and cancer samples of the training set by filtering based on two criteria: *p* value < 0.05 and fold change ≥ 2. The intersection of DEGs and calcium-related genes was analyzed by the Kyoto Encyclopedia of Genes and Genomes (KEGG) pathway, GO functional enrichment and protein–protein interaction.

### 2.2. Construction of the Prognostic Model

A univariate Cox regression analysis was conducted to identify the calcium-related DEGs associated with patient survival in the training set. A *p* value of 0.01 was selected as the significance threshold. Subsequently, calcium-related DEGs related to overall survival (OS) were analyzed by least absolute shrinkage and selection operator (LASSO) regression to narrow the range. A multivariate Cox regression analysis was then performed to develop a calcium-related risk signature based on the training cohort. The risk score was calculated as follows: Riskscore = ∑^N^_i = 1_^(Coefi,×, Expi)^, where Exp_i_ is the expression level of calcium-related genes and *Coef_i_* is the corresponding regression coefficient of the genes calculated by a multivariate Cox regression analysis.

### 2.3. Validation of the Prognostic Model

By using the median risk score as the cutoff point, patients in the training cohort, testing cohort and total cohort were further subdivided into high-risk and low-risk groups. Then, time-dependent receiver operating characteristic (ROC) curves, Kaplan–Meier survival analysis and prognostic nomograms were established to verify the efficacy of the risk model in the training cohort, testing cohort and total cohort. An area under the curve (AUC) > 0.7 was considered acceptable. Patients with different ages, grades, stages and peritoneal cytology were subdivided into high-risk and low-risk groups. Then, OS was compared between the two groups to explore whether the risk model had an independent predictive value.

### 2.4. Functional Enrichment Analysis between Different Risk Groups

DEGs between the high- and low-risk groups were obtained by the limma package. GO, enrichment analysis and GSEA were applied to explore the potential pathways.

### 2.5. Cell Culture

Endometrial cancer cell lines, Ishikawa, HEC-108 and AN3CA were purchased from the American Type Culture Collection cell bank and were experimentally preserved by the Department of Gynecology, Peking University People’s Hospital. Endometrial cancer cell lines were obtained from ATCC. Ishikawa and HEC-108 cell lines were cultured in DMEM-F12 mediums (SH30023.01, HyClone, Logan, UT, USA) containing 10% fetal bovine serum (ExCell Bio, Shanghai, China). AN3CA cells were maintained in MEM mediums (CM50011, Macgene, Biotech Co., Ltd., Beijing, China). After the cell confluence reached 80%, the cells were digested into a single-cell suspension for subsequent experiments.

### 2.6. Western Blotting

The cells were treated with a RIPA lysis buffer containing PMSF and a phosphatase inhibitor (Beijing Pulley). A total of 15 μg of the lysates was added to the gel (Biotides, Beijing) and separated after electrophoresis. Then, the proteins were transferred to the NC membranes. Next, 5% milk was used for blocking for one hour. Anti-CACNA2D1 (27453-1-AP, Proteintech, Rosemont, IL, USA) and GAPDH (30203ES50, Yeasen, China) were diluted with antibody diluents at 1:1000 and 1:5000, respectively, and incubated overnight at 4 °C. After washing the NC membrane, the secondary antibody was subsequently incubated. The proteins were detected by a ChemiDoc imaging system (Bio-Rad, Hercules, CA, USA).

### 2.7. Immunofluorescence Assay

To determine the expression of CACNA2D1, the cells were fixed and blocked with 3% BSA for 30 min. Then, the cells were incubated overnight with the CACNA2D1 antibody (27453-1-AP, Proteintech, Chicago, IL, USA) (1:200) and TRITC phalloidin (40734ES75, Yeasen, Shanghai, China) at 4 °C. The cells were incubated with a fluorescent secondary antibody for 60 min. Images were obtained using a confocal microscope.

### 2.8. siRNA-Induced Gene Silencing

SiRNA sequences were utilized in cells to generate *CACNA2D1* gene silencing. SiRNA targeting the *CACNA2D1* gene and a negative control were obtained from Hanheng Biotechnology (Shanghai, China). The transfection was performed by Lipo3000 (Invitrogen, Carlsbad, CA, USA) according to the manufacturer’s instructions.

### 2.9. Real-Time Quantitative PCR

Total RNA was extracted with TRIzol™ (15596018, Invitrogen, Carlsbad, CA, USA). Reverse transcription was performed using the 1st Strand cDNA Synthesis Kit (11141ES60, Yeasen, China) and a quantitative PCR was performed using qPCR SYBR Green Master Mix (11201ES08, Yeasen, China). Primer sequences were synthesized by Tsingke Biotechnology Co., Ltd. (Beijing, China) and are summarized in Table 2. The mRNA level of the target gene was normalized using *GAPDH*.

### 2.10. Cell Counting Kit 8 (CCK8) Assay

To investigate the effects of *CACNA2D1* on cell proliferation, a negative control and *CACNA2D1* siRNA cells were plated in 96-well plates. After 0 h, 24 h, 48 h and 72 h of incubation, 10 µL of CCK-8 solution (C0005, Targetmol, USA) were added to the well. Then, the cells were incubated for 2 h and were detected by a microplate reader at a 450 nm wavelength.

To examine the effect of AMs on cell proliferation, Ishikawa cells were seeded into 96-well dishes at a density of 5 × 10^3^ cells per well. Next, the cells were treated with AM (HY-B0317, MedChemExpress, Monmouth Junction, NJ, USA) at multiple concentrations (0, 1.25, 5, 10, 12.5, 20, 25, 40, 50 µM). After 72 h of incubation, 10 µL of CCK-8 solution were added for detection.

### 2.11. EdU Staining

After transfection for 48 h, the negative control and *CACNA2D1* siRNA cells were plated in 96-well plates and were stained with EdU solution for 2 h. Then, the cells were fixed, permeabilized and incubated with an Apollp reaction solution. Hoechst 33342 was used to stain the nuclei. Finally, a Leica microscope was used to collect pictures.

To examine the effect of AMs on cell proliferation, Ishikawa cells were seeded into a 96-well plate at a density of 10,000 per well. After 12 h, AM (15 μM) was added to the well for 48 h. EdU staining was visualized using a BeyoClick™ EdU-594 kit (C0078S, Beyotime, Shangai, China).

### 2.12. Annexin V/PI Staining

Ishikawa cells were seeded into a six-well plate and were treated with DMSO, 10 μM of AM or 15 μM of AM for 12 h. Then, the cells were digested, harvested and resuspended in a 1x binding buffer. Then, 5 µL of annexin V and 5 μL of propidium iodide (PI) (556547, BD Bioscience, San Jose, CA, USA) were added to the tube. The cells were incubated for 15 min and were protected from light. Finally, the apoptotic rate was quantified by flow cytometry.

### 2.13. In Vivo Assay

Five-week-old female BALB/c nude mice were purchased from Beijing Weitong Lihua Experimental Animal Technology Co., Ltd (Beijing, China). The mice were raised and monitored under SPF conditions. Ishikawa cells (3 × 10^6^) in 100 µL were subcutaneously injected into the right armpit region. Once the tumor volume reached approximately 100 mm^3^, the mice were randomly divided into three groups with 6 mice in each group. The mice were treated with vehicle, AM (15 mg/kg) or AM (20 mg/kg) every two days. The mice were sacrificed at the end of the experiment and the tumors were removed and weighed. Moreover, the tissue samples were fixed, embedded and sectioned for further detection.

### 2.14. Immunohistochemistry

The mouse Balb/c tumor specimens were incubated with antibody against proliferating cell nuclear antigen (PCNA) (ab92552, Abcam, Cambridge, UK). For subsequent incubation with horseradish peroxidase-conjugated biotin, the tissues were incubated with a secondary biotin-coated antibody for one hour. Visualization was performed using a DAB chromogenic solution.

### 2.15. Statistical Analysis

Data are presented as the means ± standard deviation. GraphPad 8.0 (GraphPad Software, San Diego, CA, USA) and R software v4.0.5 (https://www.r-project.org/, accessed on 1 May 2022) were used for statistical analysis. Student’s *t*-test, one-way ANOVA and chi-square tests were performed. The significant difference level was set at *p* < 0.05.

## 3. Results

### 3.1. Identification of Calcium-Related Differentially Expressed Genes in EC

**Figure 1:**
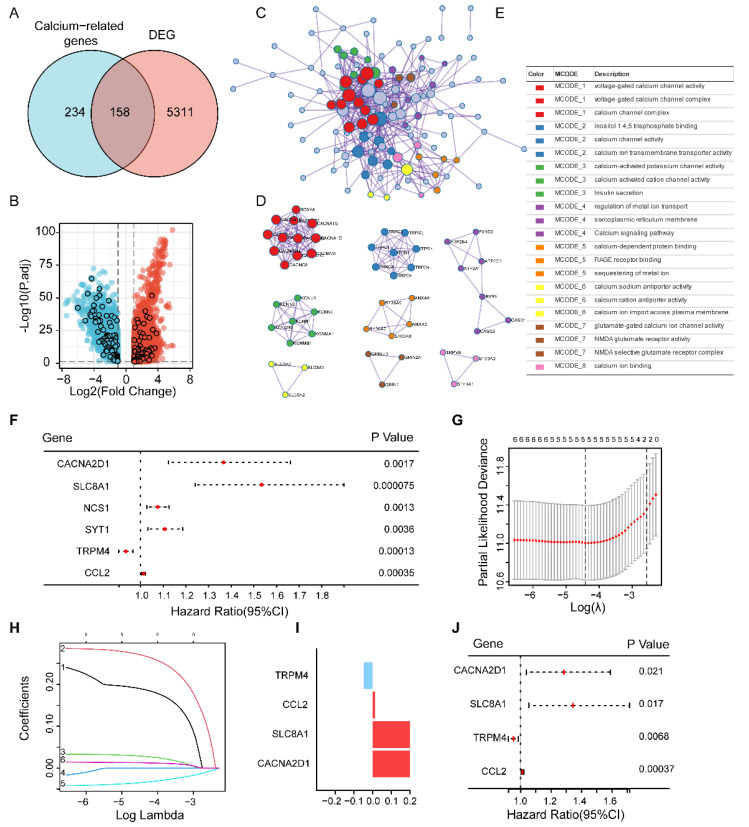
Prognostic analysis of differentially expressed calcium-related genes and the construction of a risk score signature. (**A**) Venn diagram to identify the common genes of calcium-related genes and DEGs. (**B**) Volcano plot for DEGs. A total of 158 calcium-related DEGs are highlighted in boxes. (**C**,**D**) Visualization of the protein–protein interaction (PPI) network formed by all 158 genes. The MCODE complexes are colored according to their clustering. (**E**) Significant genes of each MCODE complex are labeled and visualized. (**F**) Forest plots showing the results of the univariate Cox regression analysis of 6 prognostic genes. (**G**,**H**) The cvfit and lambda curves in LASSO regression. (**I**) The coefficients of four genes. (**J**) Forest plots showing the results of the multivariate Cox regression analysis of 4 prognostic genes.

A total of 537 cancer samples and 27 normal samples were downloaded from TCGA. The DEGs comparing cancer cases with normal cases were obtained using the limma R package. Next, we extracted 392 calcium-related protein-coding genes with relevance scores of eight from the GeneCards database (Supplementary Table S1) to create prognostic gene signatures. The intersection of DEGs and calcium-related genes revealed 158 hub genes, including 66 upregulated and 92 downregulated genes (Figure 1A,B) (Supplementary Table S2). The heatmap is shown in Supplementary Figure S1A. An analysis of the KEGG pathways indicated that the top pathway associated with 158 genes was the calcium signaling pathway (Supplementary Figure S1B,C). The GO analysis showed that the 158 hub genes linked with biological processes were mainly enriched in calcium ion transport and calcium ion homeostasis (Supplementary Figure S1D,E, Supplementary Table S3). To further explore the relationship between these calcium-related genes, an interaction network between these hub genes was mapped using Metascape. The 158 genes were mainly clustered into eight MCODE components, including “voltage-gated calcium channel”, “inositol 1,4,5 trisphosphate binding”, “calcium-activated potassium channel activity”, “sarcoplasmic reticulum membrane”, “calcium-dependent protein binding”, “calcium: sodium antiporter activity”, “glutamate-gated calcium channel” and “calcium ion binding” (Figure 1C–E).

### 3.2. Construction of the 4-Gene Risk Signature

The 537 samples were randomly divided into a training set and a testing set at a ratio of 1:1. Based on the training set, the univariate Cox regression model was employed to identify prognostic calcium-related DEGs with the threshold value of *p* < 0.01. Then, six prognostic genes were obtained for further study, including *CACNA2D1*, *SLC8A1*, neuronal calcium sensor 1 (NCS1), synaptotagmin 1 (SYT1), *TRPM4* and CC chemokine ligand 2 (CCL2). As shown in Figure 1F, *TRPM4* is a protective factor affecting the OS of EC patients, with a hazard ratio (HR) < 1. The other five genes were risk factors with HRs > 1.

Next, a least absolute shrinkage and selection operator (lasso) regression model was applied to identify prognostic calcium-related DEGs and construct the prognostic model. LASSO regression is a popular technique used in regression analysis with high-dimensional predictors [10]. After 1000 cross-validations of the parameter λ, four genes were selected, and their LASSO coefficients were obtained. The risk score of the four-gene signature was calculated using the following formula: Risk score = (0.2504 × *CACNA2D1* expression) + (0.2964 × *SLC8A1* expression) − (0.04739 × *TRPM4* expression) + (0.01346 × *CCL2* expression) (Figure 1G–I). The multivariate analysis revealed that four genes were all independent prognostic factors associated with OS in EC patients in the training set (Figure 1J, Supplementary Table S4). All these results suggest that 4 out of the 158 calcium-related DEGs were considered to have prognostic value and were used to construct a four-gene risk model, including *CACNA2D1*, *SLC8A1*, *TRPM4* and *CCL2* after univariate, LASSO regression and multivariate analyses.

### 3.3. Validation of the 4-Gene Calcium-Related Risk Signature

**Figure 2:**
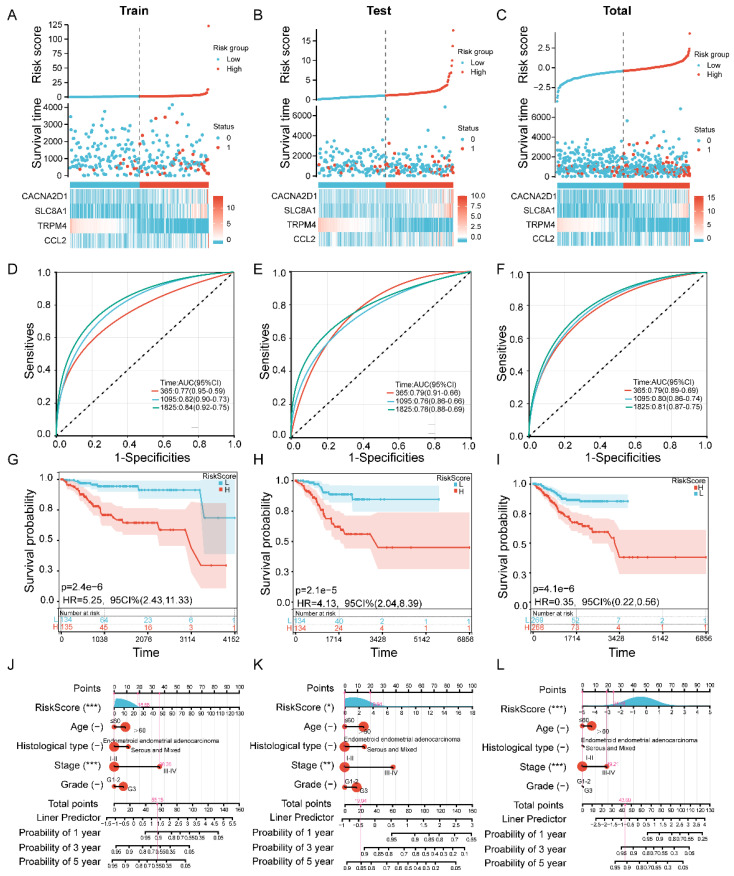
Validation of the calcium-related signature model in the training cohort, testing cohort and total cohort. (**A**–**C**) Risk curves, survival statuses and gene expression levels for high- and low-risk groups in the TCGA-training set, TCGA-test set and TCGA-total set. (**D**–**F**) ROC curves showing the potential of the prognostic 4-gene calcium-related gene model in predicting 1-, 3- and 5-year OS in the TCGA-training, TCGA-testing and TCGA-total sets. (**G**–**I**) Kaplan–Meier curves for survival status and survival time in the TCGA-training, TCGA-testing and TCGA-total sets. (**J**–**L**) The nomograms to predict the 1-year, 3-year and 5-year overall survival rates of EC patients in the TCGA-training, TCGA-testing and TCGA-total sets. * *p* < 0.05, ** *p* < 0.01 and *** *p* < 0.001.

Based on the RiskScore formula, the patients were divided into low-risk and high-risk groups using the median risk scores as the cutoff. To verify the effectiveness of the four-gene calcium-related risk signature, Kaplan–Meier survival and ROC curves were used to evaluate the accuracy of the risk model in predicting the survival of EC patients. The results indicated that the four-gene risk signature was significantly associated with prognosis and that the prognosis of the high-risk patients was significantly worse than that of the low-risk patients in the training set, testing set and total set. In addition, *CACNA2D1*, *SLC8A1* and *CCL2* had a higher expression in the high-risk groups, while TRPM4 had a lower expression in the high-risk groups. This result indicates that *TRPM4* is a factor for positive prognosis, whereas the others are factors for adverse survival (Figure 2A–C).

Next, the ROC analysis of the high-risk and low-risk groups revealed that the AUCs for 1-, 3- and 5-year OS in the TCGA-training set were 0.77, 0.82 and 0.84, respectively (Figure 2D). In the TCGA-testing set, the AUC values were 0.79, 0.76 and 0.78 at the 1-, 3- and 5-year time points, respectively (Figure 2E). In the TCGA-total set, the AUC values were 0.79, 0.80 and 0.81 at the 1-, 3- and 5-year time points, respectively (Figure 2F). Moreover, the Kaplan–Meier survival analysis showed that the survival rates of the EC patients in the high-risk groups were significantly worse than those in the low-risk groups (Figure 2G–I).

To further explore whether the four-gene calcium-related risk model is an independent prognostic factor, we conducted both univariate and multivariate Cox regression analyses in the TCGA-training set, TCGA-testing set and TCGA-total set. The univariate analysis suggested that RiskScore, stage, grade and histological type were significant prognostic factors in the three sets (Supplementary Tables S5–S7). Interestingly, the RiskScore and stage were also independent prognostic factors in the three sets (Supplementary Tables S5–S7). Based on our Cox regression analysis, we established a nomogram to directly calculate the survival rate of each EC patient by integrating clinical factors (age, histological type, stage and grade) and RiskScore. According to the established nomogram, each patient had a total score by adding up the points of the above prognostic variables. A calibration curve for the nomogram’s prediction of 3- and 5-year overall survival performed exceptionally well in the TCGA-training set, TCGA-testing set and TCGA-total set (Figure 2J–L). These results suggested that the four-gene risk model predicts the survival of EC patients with a high accuracy.

### 3.4. Relationship between the 4-gene Calcium-Related Signature and the Clinicopathological Characteristics in EC Patients

**Figure 3:**
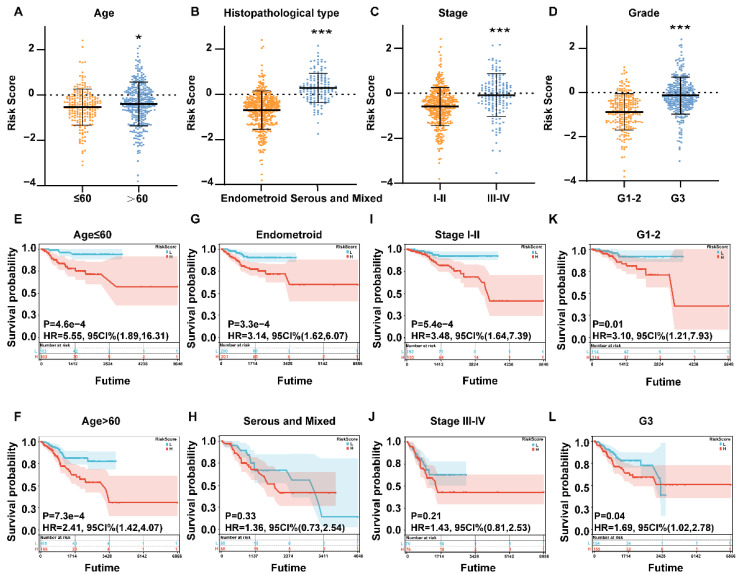
Correlation analysis between the prognostic signature and different clinicopathological characteristics in the TCGA-total cohort. (**A**–**D**) The histogram depicting the significant differences in the RiskScores in EC patients stratified by age, histopathological type, stage, and grade. (**E**,**F**) Subgroup analysis of Kaplan–Meier curves in different ages ≤60 and >60. (**G**,**H**) Subgroup analysis of Kaplan–Meier curves in different histopathological types. (**I**,**J**) Subgroup analysis of Kaplan–Meier curves in different stages I–II and III–IV. (**K**,**L**) Subgroup analysis of Kaplan–Meier curves in different grades 1–2 and 3. * *p* < 0.05, and *** *p* < 0.001.

To further investigate the relationship between the RiskScore and clinical characteristics, the samples in the TCGA-total set were divided into subgroups and the RiskScore was calculated. Interestingly, there were significant differences in age (*p* < 0.05), histopathological type (*p* < 0.001), stage (*p* < 0.001) and grade (*p* < 0.01). The subgroup with old age, advanced stages and grades exhibited a higher RiskScore. In addition, patients with serous and mixed histopathological types had higher RiskScores than those diagnosed with endometroid type (Figure 3A–D). In addition, the subgroup analysis grouped by age of 60, histopathological type, grade and stage revealed that patients of all ages, endometroid type, stage I–II and all grades with a higher score had a poor prognosis, suggesting the accuracy and efficiency of this constructed prognostic model (Figure 3E–L). These results indicate that the four-gene calcium-related risk model has good potential for predicting the prognosis of most EC patients.

### 3.5. Functional Enrichment of the 4-Gene Risk Model

**Figure 4:**
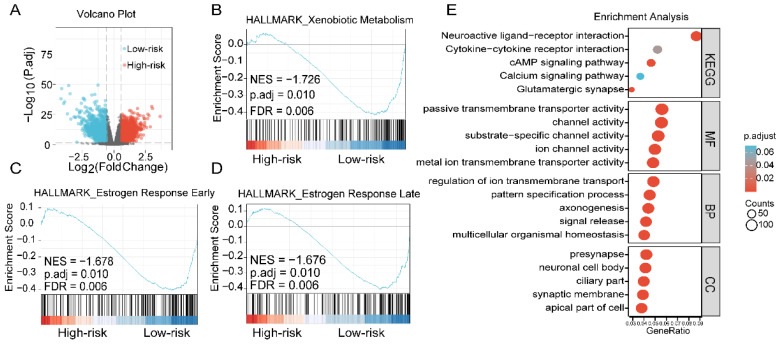
Enrichment analysis of the high-risk group and low-risk group based on the 4-gene prognostic signature. (**A**) Volcano map of DEGs between the high-risk and low-risk groups. (**B**–**D**) GSEA against Hallmark showing significant enrichment of xenobiotic metabolism, estrogen response early and estrogen response late, respectively, in the low-risk endometrial cancer patients. (**E**) KEGG and GO analyses showing that calcium-related biological processes were significantly enriched.

DEGs between the high-risk group and the low-risk group of the TCGA-total set were identified by the cutoff criteria |FC| > 1.5 and FDR < 0.05, shown in a volcano plot (Figure 4A). Then, 2888 DEGs were obtained for further study (Supplementary Table S8). To explore the potential pathways between the different risk groups divided by the four-gene prognostic signature, a GO, KEGG pathway analysis and GSEA of 2888 DEGs were performed. DEGs with FDR < 0.05 were included in the GSEA to comprehensively investigate the key pathway between the high- and low-risk groups. The GSEA showed that DEGs of the high-risk and low-risk groups were enriched in late/early estrogen response signaling and xenobiotic metabolism (Figure 4B–D). KEGG enrichment revealed that calcium signaling, cAMP signaling and cytokine–cytokine receptor interactions were enriched. In GO function (BP, CC, MF) enrichment, the regulation of ion transmembrane transport, ciliary part and ion channel activity were enriched (Figure 4E, Supplementary Table S9). These data revealed the relation of DEGs with cancer-related processes and hormone signaling. In summary, the RiskScore of the four-gene risk model is mainly related to calcium signaling, estrogen response signaling and ion channel activity in EC, implying its specificity in endometrial cancer.

Next, we analyzed the association between the four genes and four clinicopathological factors (histological type, stage, grade and tumor invasion). The results showed that *CACNA2D1* expression was associated with all four clinicopathological factors (*p* < 0.05) (Supplementary Figure S2A–D). High *SLC8A1* expression was significantly correlated with the serous/mixed type and a higher stage and grade (Supplementary Figure S2E–G). However, there were no apparent associations between *SLC8A1* expression and tumor invasion (Supplementary Figure S2H). In contrast, *TRPM4* expression was positively correlated with all four clinicopathological factors, whereas *CCL2* expression showed no relationship with any parameters (Supplementary Figure S2I–P). These results indicated that *CACNA2D1* and *TRPM4* mediate EC progression. Our previous study identified *TRPM4* as an independent prognostic factor. *TRPM4* deletion significantly promoted growth and migration in EC cells [11]. *CACNA2D1* has been reported to have potential as a therapeutic target for gastric cancer [12], lung cancer [13] and epithelial ovarian cancer [14]. However, the function of *CACNA2D1* remains unclear. Moreover, *CACNA2D1* was a risk factor in Cox regression analysis and exhibited a high coefficient in our risk model. Therefore, we investigated the influence of *CACNA2D1* on EC cell behavior.

### 3.6. Knockdown of CACNA2D1 Inhibited the Proliferation and Migration of EC Cells

**Figure 5:**
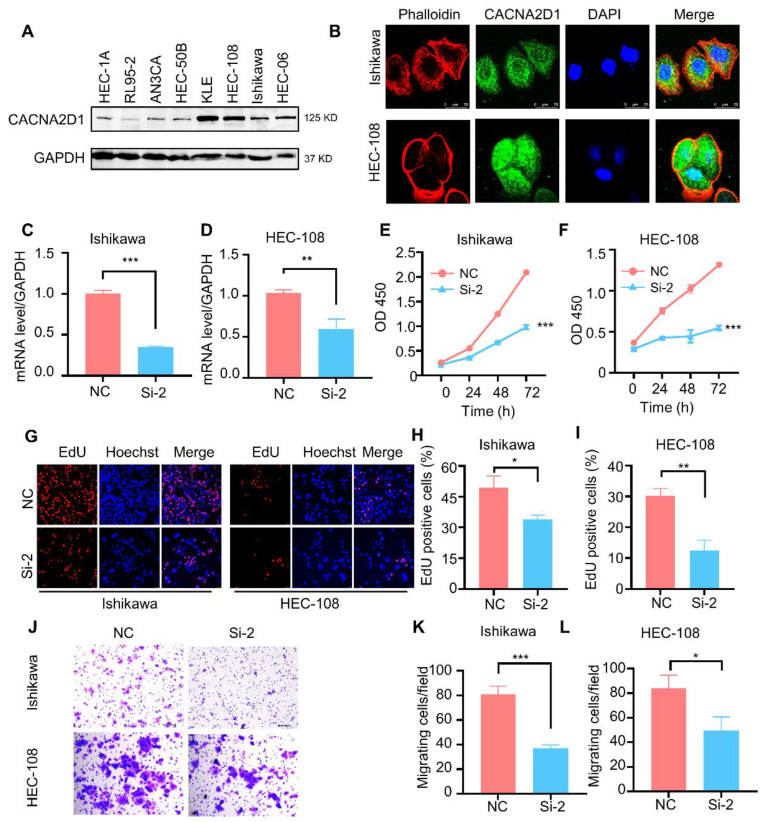
Silencing *CACNA2D1* inhibited the proliferation and migration of EC cells. (**A**) Representative images of Western blotting of endogenous expression of CACNA2D1 in eight endometrial cancer cell lines. (**B**) Immunofluorescence staining of CACNA2D1 and phalloidin in Ishikawa (**upper**) and HEC-108 (**lower**) cells. Nuclei were stained with DAPI. Scale bar, 25 µm (Magnification: 40×). (**C**,**D**) Transfection efficiency of *CACNA2D1* via small interfering RNA in Ishikawa (**C**) and HEC-108 cells (**D**). (**E**,**F**) CCK8 assay showing the effect of CACNA2D1 silencing on Ishikawa (**E**) and HEC-108 (**F**) cell proliferation. (**G**) EdU staining showing the effect of *CACNA2D1* silencing on Ishikawa and HEC-108 cells. (**H**,**I**) The statistical data of the EdU-positive rate after interfering with *CACNA2D1*. (**J**) Transwell assay detecting the effect of CACNA2D1 silencing on Ishikawa and HEC-108 cells. (**K**,**L**) The statistical data of the migrating cells after interfering with *CACNA2D1*. Data are shown as the mean ± SD. T-test. * *p* < 0.05, ** *p* < 0.01 and *** *p* < 0.001. These experiments were conducted in triplicate independently with similar results.

First, endogenous CACNA2D1 expression was examined by Western blotting in eight endometrial carcinoma cell lines, among which the HEC-108, KLE, Ishikawa and HEC-06 cell lines showed relatively higher levels of CACNA2D1 than the other four EC cell lines (Figure 5A). Second, immunofluorescence staining revealed that CACNA2D1 was mainly localized in the cytoplasm of the HEC-108, Ishikawa and AN3CA cells (Figure 5B, Supplementary Figure S3A). The HEC-108 and Ishikwa cell lines were chosen for further study.

Third, we investigated the biological function of *CACNA2D1* by knocking down *CACNA2D1* using siRNA in vitro. RT–PCR and Western blotting were performed to confirm *CACNA2D1* silencing by comparison with the corresponding negative control (NC). Si-2 was selected for subsequent experiments for its highest knockdown efficiency (Figure 5C,D, Supplementary Figure S3B,C). CCK8 and EdU assays revealed an obvious inhibition in cell proliferation in response to *CACNA2D1* knockdown (Figure 5E–I). Furthermore, a cell cycle analysis showed that *CACNA2D1* knockdown decreased the proportion of cells in the S phase but increased the proportion of cells in the G1 phase in Ishikawa cells (Supplementary Figure S3D,E). These results suggest that *CACNA2D1* increased cellular proliferation by promoting cell cycle progression. In addition, the Transwell migration assay showed that *CACNA2D1* knockdown inhibited the migration of Ishikawa and HEC-108 cells (Figure 5L–N). In summary, *CACNA2D1* facilitated EC progression.

### 3.7. The Calcium Channel Blocker AM Inhibited Cancer Progression In Vitro and In Vivo

**Figure 6:**
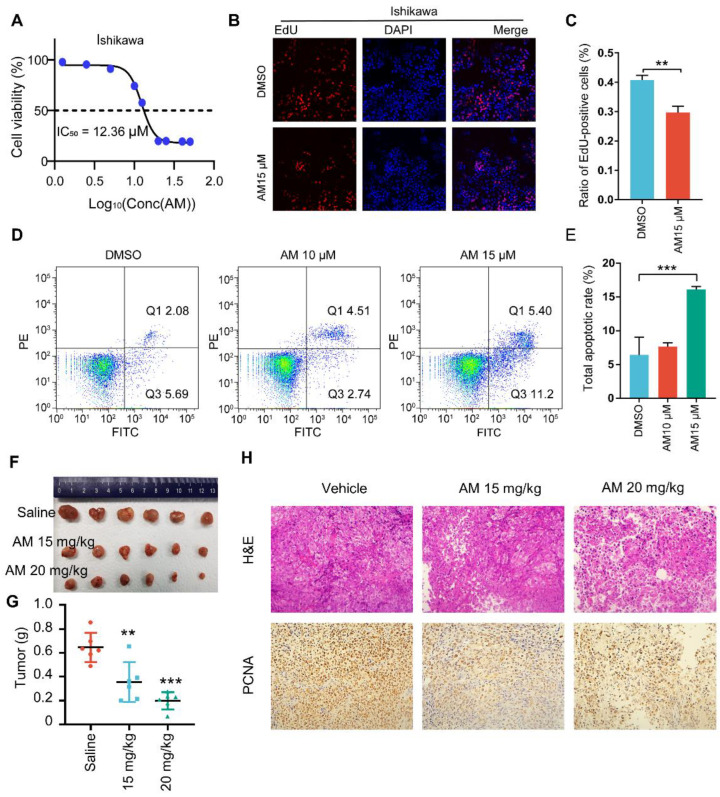
AM inhibited EC cell proliferation both in vitro and in vivo. (**A**) A CCK-8 assay was used to detect the effect of AMs on the proliferation of Ishikawa cells. (**B**) EdU staining showing the effect of AMs on Ishikawa cells. (**C**) The statistical data of the EdU-positive rate after treatment with AMs. (**D**) Cell apoptosis rate was detected by flow cytometry using Annexin-V/PI staining. (**E**) Representative images of tumor samples (*n* = 6). (**F**,**G**) The statistical data of tumor weight are shown as the mean ± SD. One-way ANOVA. ** *p* < 0.01, *** *p* < 0.001. (**H**) H&E and PCNA staining of sections from mice treated with AMs (0, 15, 20 mg/kg). Data are shown as the mean ± SD. (**C**) *t*-Test. (**E**) One-way ANOVA. ** *p* < 0.01 and *** *p* < 0.001. These experiments were conducted in triplicate independently with similar results.

Amlodipine (AM) is a clinically used antihypertensive drug. The Therapeutic Target Database (TTD; https://db.idrblab.org/ttd/, accessed on 1 May 2022) identified AM as a specific blocker of CACNA2D1 [15]. Thus, we assessed the function of *CACNA2D1* using AM. Ishikawa cells were seeded in 96-well plates, and a series of concentration gradients of AMs were added to the plates. The 50% inhibitory concentration (IC_50_) value (IC_50_) of Ishikawa cells at 72 h was 12.36 μM (Figure 6A). In addition, the EdU staining assay revealed that the EdU-positive rate of the AM-treated Ishikawa cells was significantly reduced compared with that of the control group (Figure 6B,C). These results suggest that the AMs inhibited EC cell proliferation. Moreover, Annexin V/PI staining was performed to explore the effects of AM on cell apoptosis. The results showed that the apoptotic rate of the AM-treated (15 μM) Ishikawa cells was 16.8%, which was 2.16-fold higher than that in cells treated with DMSO (Figure 6D).

Next, we established a subcutaneous transplanted tumor model in Balb/c mice by injecting Ishikawa cells. A peritoneal injection of AM (0, 15 or 20 mg/kg) was started on day 7 and ended on day 21 after tumor inoculation. The mice were all sacrificed simultaneously after the treatment was completed. Images of the tumors revealed that the AM treatment inhibited tumor growth in vivo in a dose-dependent manner (Figure 6E,F). Hematoxylin-eosin (H&E) staining showed that tumor tissue in the AM-treated groups had large necrosis and an unclear cell structure. Furthermore, proliferating cell nuclear antigen (PCNA) staining was applied to indicate active proliferation. The results showed that PCNA-positive cells were significantly decreased after treatment with AMs, indicating less proliferation of the EC cells, which was consistent with the H&E staining results (Figure 6G). These results suggest that AM exhibits significant potential in the treatment of EC.

## 4. Discussion

Endometrial cancer is the fourth most common cancer in women worldwide, with a constantly increasing incidence [1]. In the last 30 years, the overall incidence has risen by 132% [16]. In addition, epidemiological data show that EC mortality has decreased at the global level but has increased in more than 40% of countries. An increasing number of women are now dying from EC and EC-related complications, which highlights the urgency for prognostic indictors and novel treatments [17]. Calcium ions are the second messenger of cell transduction and play an important role in various cellular processes. It is also a key part of hormone signaling. However, in endometrial cancer, a hormone-responsive tumor, there are few studies on calcium. At present, it was reported that in a variety of tumor cells, such as breast cancer [18], lung cancer [19] and gastrointestinal cancer [20], the aberrant expression of transmembrane calcium channel proteins leads to abnormal calcium flux in tumor cells, subsequently remodeling the downstream signaling pathway. Meanwhile, calcium ions may play an important role in apoptosis, migration and immune escape [21,22,23]. Ca^2+^ has been considered a promising predictive biomarker in many cancers, such as breast cancer, ovarian cancer and EC [4,24,25]. Earlier studies have reported that a higher adjusted calcium level was associated with a poor prognosis in bladder cancer [26] and prostate cancer [27]. Our previous analysis suggested that serum calcium was an independent risk factor for lymph node metastasis in EC patients [24]. Therefore, targeting calcium may become a new approach in the management of tumors. However, it remains unclear which calcium-related genes act as prognostic biomarkers.

The highlight of this study was identifying prognostic calcium-related genes in endometrial cancer. The nomogram combining age, stage, grade and risk score may comprehensively evaluate the risk of patients in clinical practice. A univariate Cox regression analysis identified six genes that were significantly associated with the OS of EC patients. Then, a LASSO regression and multivariate Cox regression analysis were performed to select four key calcium-related genes (*CACNA2D1*, *SLC8A1*, *TRPM4* and *CCL2*) to construct the model. *CACNA2D1*, *SLC8A1* and *TRPM4* are calcium channels or exchangers that directly regulate cytoplasmic calcium concentrations, thus influencing calcium homeostasis. *CACNA2D1* is one of the genes encoding the α2δ subunit family of voltage-gated calcium channels (VGCCs). *SLC8A1*, a plasma-membrane Ca^2+^ exchanger, mediates Ca^2+^ efflux or influx in exchange for Na^+^ and modulates cellular functions, including cell apoptosis, proliferation and mitochondrial activities [28,29]. It was identified as a mechanical stimulus-related gene that could regulate the cytoskeleton and subsequent cell migration in endometrial cancer [9]. *TRPM4* is a nonselective cation channel that is permeable to Ca^2+^ and affects calcium homeostasis. Our study considered *TRPM4* a protective prognostic gene in endometrial cancer, which was consistent with our previous report [11]. However, it was identified as a risk factor for poor survival in prostate cancer [30], breast cancer [31] and diffuse large B-cell lymphoma [32]. *CCL2*, also known as monocyte chemoattractant protein 1 (MCP-1), belongs to the CC-chemokine family of substances. It activates chemotactic activity and triggers calcium influx by binding to its receptor, C-C motif chemokine receptor-2 (CCR2) [33]. An analysis on TCGA data showed that higher expression levels of *CACNA2D1* and *SLC8A1* were associated with EC progression and indicated a worse prognosis. *CACNA2D1* downregulation was reported to induce the erythroid differentiation of chronic myeloid leukemia cells, indicating that *CACNA2D1* might be associated with higher cell stemness [34]. In addition, *CACNA2D1* was identified as a surface marker of cancer stem cells in liver cancer [35,36] and breast cancer [37]. A high expression of CACNA2D1 was associated with a poor prognosis in several cancers, including ovarian cancer [14], lung cancer [38], hepatocellular cancer [39] and gastric cancer [12]. In our study, we verified the function of *CACNA2D1* in EC for the first time. It may be a biomarker and target of therapy in EC.

In fact, there is a long history of discussion of the relationship between CCB use and the increased risk of tumorigenesis and progression. AM has exhibited antitumor activity in epidermoid carcinoma [40], lung cancer [41], gastric cancer [42] and breast cancer [43]. In this study, we also explored the potential of AM in treating EC. However, despite the proven efficacy of the treatment, CCBs used as anticancer therapeutic agents suffer from some limitations. One of the important concerns is the indication. AM might be particularly suitable for patients with cancer and hypertension. The dose of AM is another concern. Previous studies have shown that the dose of AM for cancer treatment is generally higher than that for hypertension, which may limit its clinical utility for cancer therapy. Based on these considerations, we will collect clinical information and analyze the correlation between AM use and progression. In addition, nanoenabled drug delivery systems may be a new direction.

## 5. Conclusions

Taken together, this study identified four calcium-related genes via univariate Cox analysis, LASSO analysis and multivariate Cox analysis and established a predictive model for EC patients. In the TCGA-total set, the OS of patients in the high-risk group associated with age, endometroid type, stage I-II and all grades was obviously worse than that in the low-risk group. The CACNA2D1 blocker AM inhibited cell proliferation in vitro and in vivo. Our study showed that calcium homeostasis represented a considerable factor involved in the progression of EC. More importantly, it provides a new therapeutic therapy by targeting calcium or calcium channels for EC treatment.

## Figures and Tables

**Table 1:** Demographic and clinical characteristics of the TCGA-training, testing and total cohorts.

Characteristics	TCGA-Training (*n* = 269)	TCGA-Testing (*n*= 268)	Total (*n* = 537)	*p* Value
OS				
Mean ± SD	1103.08 ± 866.17	1166.20 ± 934.32	1134.58 ± 900.54	
Status				0.98
Alive	226 (84.01%)	224 (83.58%)	450 (83.80%)	
Dead	43 (15.99%)	44 (16.42%)	87 (16.20%)	
Age				
Mean ± SD	64.51 ± 11.25	63.28 ± 10.81	63.90 ± 11.04	
Histological_type				0.59
Endometrioid endometrial adenocarcinoma	205 (76.21%)	196 (73.13%)	401 (74.67%)	
Mixed	9 (3.35%)	13 (4.85%)	22 (4.10%)	
Serous	55 (20.44%)	59 (22.02%)	114 (21.23%)	
Stage				0.83
I	167 (62.08%)	167 (62.31%)	334 (62.20%)	
II	24 (8.92%)	27 (10.07%)	51 (9.50%)	
III	65 (24.16%)	58 (21.64%)	123 (22.91%)	
IV	13 (4.83%)	16 (5.97%)	29 (5.40%)	
Grade				0.45
High Grade	4 (1.49%)	7 (2.61%)	11 (2.05%)	
G1	46 (17.10%)	52 (19.40%)	98 (18.25%)	
G2	66 (24.54%)	53 (19.78%)	119 (22.16%)	
G3	153 (56.88%)	156 (58.21%)	309 (57.54%)	

**Table 2:** Primers used for the real-time PCR assay.

Genes	Species	Primers (FW)	Primers (RW)
*CACNA2D1*	Human	ACGCAGCAGTCCATATTCCTA	GCCACAATAATGAAGGGTCTTCC
*GAPDH*	Human	GGAGCGAGATCCCTCCAAAAT	GGCTGTTGTCATACTTCTCATGG

## Data Availability

Data presented in this study are contained within this article and in the supplementary materials, or are available upon request to the corresponding author.

## Supplementary Materials

The following supporting information can be downloaded at: https://www.mdpi.com/article/10.3390/cells11193156/s1, Figure S1: The expression and functional enrichment analysis of 158 calcium-related DEGs. (A) Heatmap of 158 calcium-related DEGs; (B,C) KEGG analysis of 158 calcium-related DEGs, with only the top 10 terms shown; (D,E) GO analysis of 158 calcium-related DEGs, with the top 10 terms of biological process (BP), cellular component (CC) and molecular function (MF) shown. Figure S2: The relationship between clinicopathologic factors (histologic type, clinical stage, histologic grade and tumor invasion) and 4 genes. (A–D) CACNA2D1; (E–H) SLC8A1; (I–L) TRPM4; (M–P) CCL2. Figure S3: Knockdown of CACNA2D1 induced cell cycle arrest. (A) Immunofluorescence staining of CACNA2D1 and phalloidin in AN3CA cells. Nuclei were stained with DAPI. Scale bar, 25 µm (Magnification: 40×); (B,C) Representative Western blotting images showing CACNA2D1 knockdown at the protein level in Ishikawa and HEC-108 cells; (D,E) Cycle distribution analysis of Ishikawa cells with NC or knockdown of CACNA2D1 expression was performed by flow cytometry. Table S1: Calcium-related genes with relevance scores of 8 from the GeneCards database. Table S2: Calcium-related DEGs of endometrial cancer. Table S3: GO analysis of 158 calcium-related DEGs. Table S4: Multivariate analysis of 4 calcium-related DEGs. Table S5: Univariate and multivariate Cox regression analyses on the association between clinicopathological factors (including the risk signature) and OS of patients in the TCGA-training dataset. Table S6: Univariate and multivariate Cox regression analyses of the association between clinicopathological factors (including the risk signature) and OS of patients in the TCGA-test dataset. Table S7: Univariate and multivariate Cox regression analyses on the association between clinicopathological factors (including the risk signature) and OS of patients in the TCGA-total dataset. Table S8: DEGs between the high-risk and low-risk groups in the TCGA-total set. Table S9: Top 5 terms of each category after KEGG and GO analysis.

## Abbreviations

EC: endometrial cancer; TCGA: The Cancer Genome Atlas; LASSO: least absolute shrinkage and selection operator; DEGs: differentially expressed genes; GO: gene ontology; GSEA: gene set enrichment analysis; AM: amlodipine; LNM: lymph node metastasis; LVSI: lymph vascular space invasion; CACNA1D: calcium voltage-gated channel subunit alpha1 D; TRPV4: transient receptor potential vanilloid 4; SLC8A1: solute carrier family 8, member 1; CCB: calcium channel blocker; KEGG: Kyoto Encyclopedia of Genes and Genomes; OS: overall survival; ROC: receiver operating characteristic; AUC: area under the curve; CCK8: Cell Counting Kit 8; NCS1: neuronal calcium sensor 1; SYT1: synaptotagmin 1; CCL2: CC chemokine ligand 2; PPI: protein–protein interaction; PCNA: proliferating cell nuclear antigen; VGCC: voltage-gated calcium channel; CCR2: C-C motif chemokine receptor-2; BP: biological process; CC: cellular component; MF: molecular function.
