# Supplementary material for: Calcium-Related Genes Predicting Outcomes and Serving as Therapeutic Targets in Endometrial Cancer
Source: Cells. 2022 Oct 8;11(19):3156. doi: 10.3390/cells11193156 (PMC9563405; doi:10.3390/cells11193156)
Supplement: Supplementary file 1 [file cells-11-03156-s001.zip › cells-1929514-supplementary.pdf]

Supplementary Materials

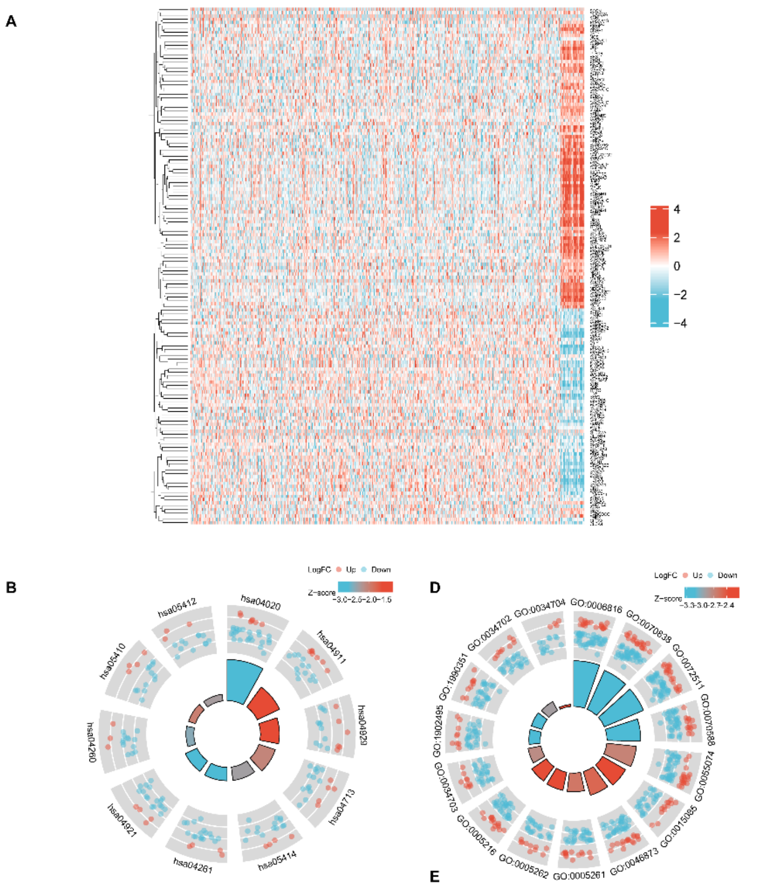

C

ID	Description
hsa04020	Calcium signaling pathway
hsa04911	Insulin secretion
hsa04929	GnRH secretion
hsa04713	Circadian entrainment
hsa05414	Dilated cardiomyopathy
hsa04261	Adrenergic signaling in cardiomyocytes
hsa04921	Oxytocin signaling pathway
hsa04260	Cardiac muscle contraction
hsa05410	Hypertrophic cardiomyopathy
hsa05412	Arrhythmogenic right ventricular cardiomyopathy

ID	Description
GO:000681	calcium ion transport
GO:007083	divalent metal ion transport
GO:007251	divalent inorganic cation transport
GO:003470	cation channel complex
GO:190249	transmembrane transporter complex
GO:003470	ion channel complex
GO:001508	calcium ion transmembrane transporte
GO:004687	metal ion transmembrane transporter a
GO:000526	calcium channel activity

Supplementary Figure S1. The expression and functional enrichment analysis of 158 calcium-related DEGs.

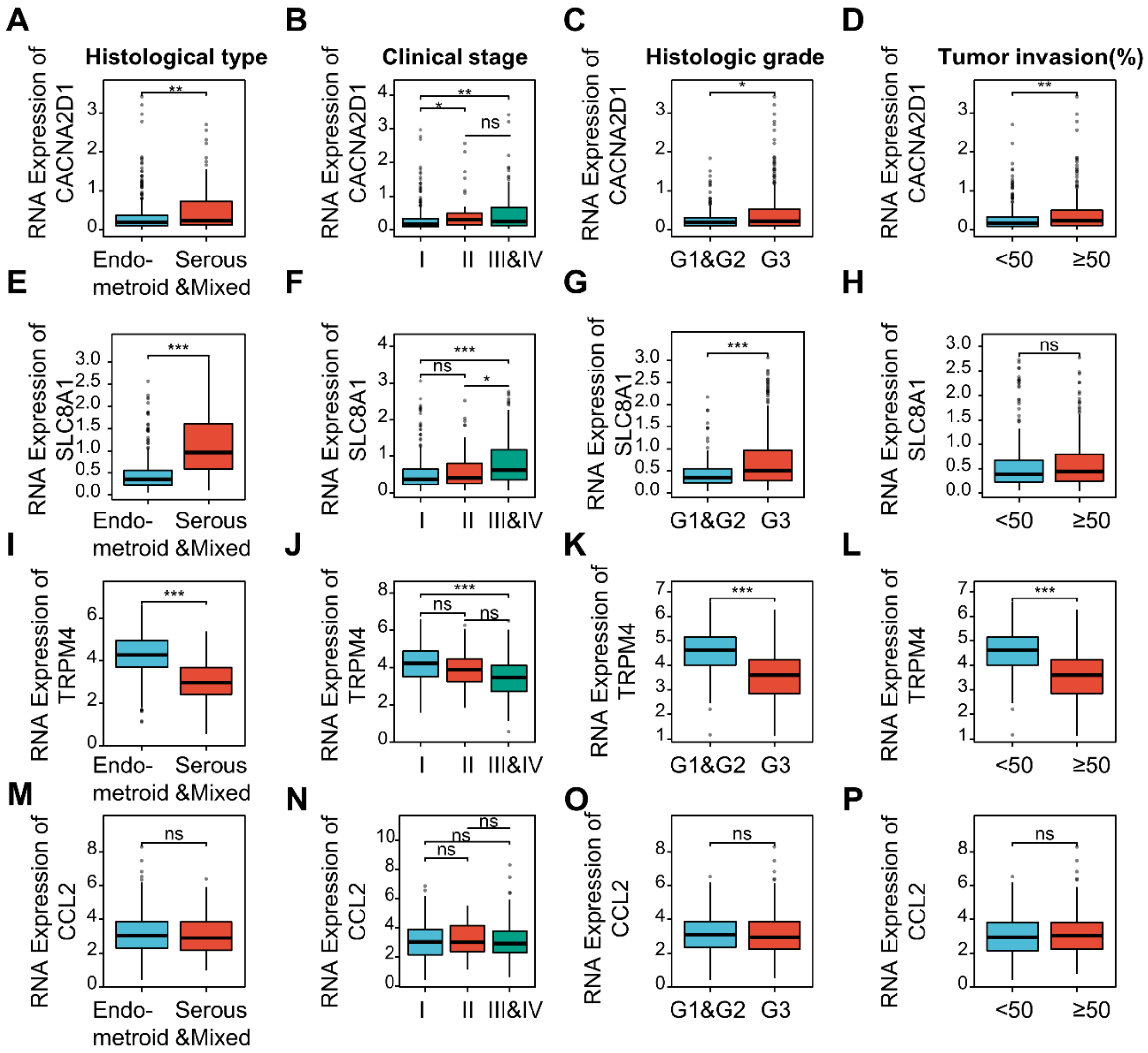

Supplementary Figure S2: The relationship between clinicopathologic factors (histologic type, clinical stage, histologic grade, and tumor invasion) and 4 genes.

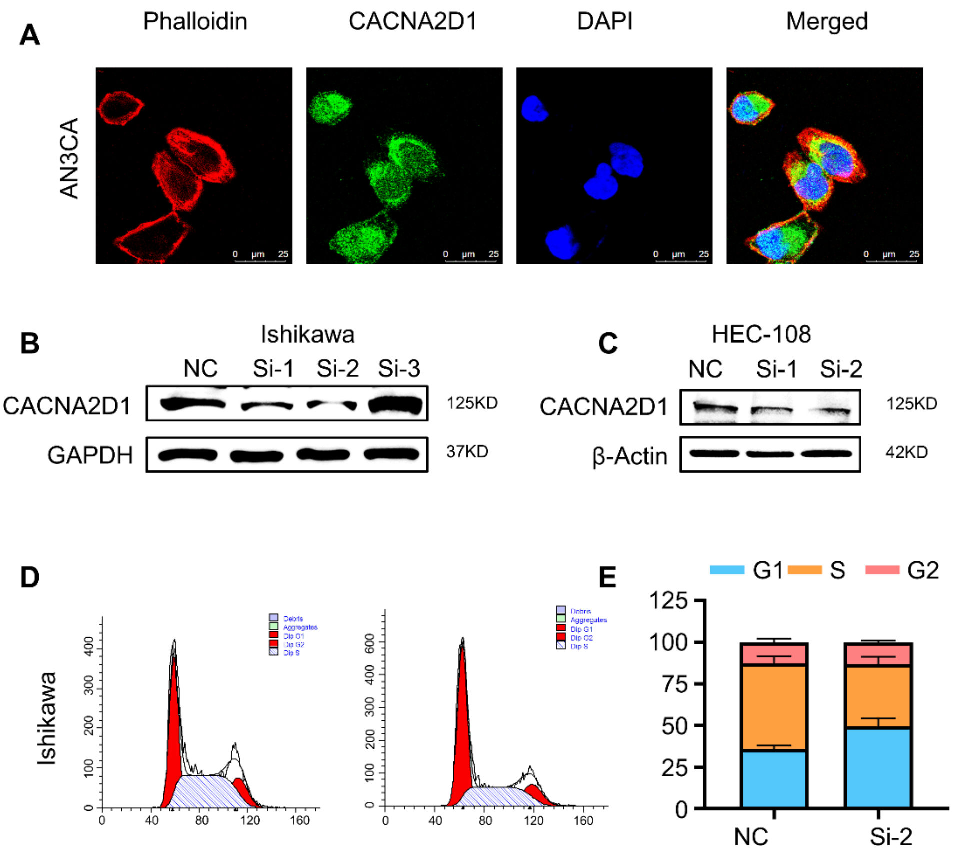

Supplementary Figure S3. Knockdown of CACNA2D1 induced cell cycle arrest.
